# Shaping national plans and strategies for rare diseases in Europe: past, present, and future

**DOI:** 10.1007/s12687-021-00525-4

**Published:** 2021-05-05

**Authors:** Victoria Hedley, Valentina Bottarelli, Ariane Weinman, Domenica Taruscio

**Affiliations:** 1 Newcastle University, Newcastle, UK; 2 EURORDIS–Rare Diseases Europe, Paris, France; 3 National Centre for Rare Diseases, Istituto Superiore di Sanità, Rome, Italy

## Abstract

Addressing the many challenges posed by rare diseases to patients, families, and society at large demands a specific national (as well as transnational) focus. Historically, the practice of elaborating and adopting national plans and strategies for rare diseases, following a request from the European Commission in 2009, has been an essential means of ensuring this focus, with 25 European Member States having adopted a plan or strategy at some stage. However, from the vantage point of late 2020, there are signs that momentum and commitment to the development, implementation, and renewal of national plans is waning, in some cases. In this article, we examine the status quo and explore the trend for national plans and strategies to expire without clear commitments or timelines for replacement. We also examine the factors and institutions which supported the initial drive towards the adoption of national plans and strategies in Europe and consider the very different climate in which the next generation of national policies may—or may not—be shaped.

## Origins of national plans and strategies for rare diseases

In the early years of the twenty-first century, European nations began to address the challenges posed by cancer by creating national action plans, as vehicles to unite efforts and give visibility to national activities and programmes (WHO 2002; Espina et al. 2018). The advantages of such policies were recognised by a community with quite different challenges but arguably an even greater need for strategic oversight and transparency at national level—the rare disease community. Rare diseases pose myriad challenges, not only to patients and families but also to professionals working with them and, by extension, to the health and social systems of each nation. In consequence of the specificities of rare diseases as a collective group (encompassing some 6–8000—most genetic—conditions), since the 1990s, rare diseases have been considered a policy priority at both Member State (MS) and—crucially—European Union level (Rodwell and Aymé 2015). The first national plan for rare diseases was adopted in France, covering the period 2005–2008. Other countries looked to this example as the benefits of a national plan or strategy for rare diseases became more apparent (Taruscio et al. 2007; Hedley et al. 2019).

The rare disease cause in Europe received a major boost in 2008 and 2009 with the publication of two policy documents: the 2008 Commission Communication ‘Rare Diseases: Europe’s challenges’ (European Commission 2008) and the Council Recommendation of 8 June 2009 on an action in the field of rare diseases (2009/C 151/02) (Council of the European Union 2009). The latter, in particular, called upon all EU MS to work collectively to pool knowledge and expertise and address some of the shared challenges around diagnostics, treatment, care and research, collaboratively—as befits a field in which cross-border cooperation is a necessity, not merely a benefit. A particular focus of pan European collaboration was the national plans and strategies agenda. The Council Recommendation of 2009 issued a specific and time-bound request to MS, which actually formed the basis of the first Theme of the Recommendation: MS were asked to:

*“Establish and implement plans or strategies for rare diseases at the appropriate level or explore appropriate measures for rare diseases in other public health strategies, in order to aim to ensure that patients with rare diseases have access to high-quality care, including diagnostics, treatments, habilitation for those living with the disease and, if possible, effective orphan drugs”*

Four sub-requests followed, the first of which was for MS to:

*“(a) elaborate and adopt a plan or strategy as soon as possible, preferably by the end of 2013 at the latest, aimed at guiding and structuring relevant actions in the field of rare diseases within the framework of their health and social systems;”*

In this way, a clear target was set. It is notable too that the Council Recommendation placed emphasis not only on the health aspects but also the social aspects of rare diseases, highlighting ‘habilitation’ alongside diagnostics and treatment.

## The realisation of cross-country support and knowledge exchange

At the time of adoption of the Council Recommendation, only 5 EU MS had adopted a national plan or strategy for rare diseases: France, Bulgaria, Greece, Portugal, and Spain (Rodwell and Aymé 2015; Hedley et al. 2019). The benefits of sharing experiences and albeit informally, benchmarking progress, were recognised, and two major avenues of opportunity were established to enable this.

The first concerns project-based support and capacity building, in the form of the EUROPLAN project (see project summary https://webgate.ec.europa.eu/chafea_pdb/health/projects/2007119/summary) and the EUCERD Joint Action: Working for Rare Diseases. EUROPLAN came first, funded through DG SANCO (as it was at the time). EUROPLAN ran from 2008 to 2011, thus anticipating the contents of the then-imminent Council Recommendation. With a European Commission budget of € 642,150, the project was coordinated by the Italian National Institute of Health-Italian National Centre for Rare Diseases. It involved 57 associated and collaborating partners from 34 countries, including EURORDIS (Rare Diseases Europe). EUROPLAN developed tools to develop and implement national plans and strategies by combining action at the European level on ‘what works’ with national foci to support efforts on the ground, as it were (Taruscio et al. 2014a). By 2011, despite a robust start, progress in the majority of countries remained elusive, and thus EUROPLAN activities continued under the EUCERD Joint Action (Lynn et al. 2017; Hedley et al. 2018), which had a mandate to assist the European Commission in the formulation and implementation of activities within the rare disease community, and to foster exchanges of relevant experience and policies and practices between MS and stakeholders. One of the Joint Action’s five major areas of activity concerned ‘the implementation of plans and strategies for rare diseases at the national level’ and the work advanced here was essentially a continuation of the EUROPLAN venture (Lynn et al. 2017).

A major focus on EUROPLAN activities was the organisation and delivery of national EUROPLAN Conferences, in which partner EURORDIS (Rare Diseases Europe) played a key role. Forty such conferences were held between 2008 and 2015 (and a further 19 conferences or roundtables under the subsequent Joint Action for rare diseases, RD-ACTION, which similarly retained a connection, in this strand of activity, to the EUROPLAN brand). The conferences were organised by National Alliances for Rare Diseases in conjunction with EURORDIS to ensure a truly patient-centred approach and bring all relevant stakeholders around the same table. These conferences were key to discuss national specific needs as well as integration of European support policies/recommendations for rare diseases in line with the Council Recommendation:

“*(d)[Countries should] take note of the development of guidelines and recommendations for the elaboration of national action for rare diseases by relevant authorities at national level in the framework of the ongoing european project for rare diseases national plans development (EUROPLAN) selected for funding over the period 2008-2011 in the first programme of Community action in the field of public health”*

A major source of guidance—to be deployed via EUROPLAN and the Joint Actions—was the Expert Groups established at the European level, which constituted the second main source of support in the goal of elaborating and adopting national plans and strategies for rare diseases. The EUCERD (EU Committee of Experts on Rare Diseases) (2010–2013) provided a dedicated space for MS representatives, patients, Industry, and independent experts to join the European Commission in exploring avenues for cross-country collaboration around many diverse aspects of the broad ‘rare disease’ topic (Aymé and Rodwell 2014). It was succeeded by the Commission Expert Group on Rare Diseases (2014-2016), with similar mandate and membership to the EUCERD (key differences included a revised category of membership, namely ‘European associations of producers of products or service providers relevant for patients affected by rare diseases’ and the fact that this body was chaired by the European Commission directly). Supported in their activities by two dedicated EU Joint Actions (the aforementioned EUCERD JA and RD-ACTION), the expert groups facilitated multidisciplinary debate and research around the problems facing each nation. Solutions were proposed, most prominently in the form of 8 sets of topically oriented recommendations representing high-level (‘soft law’) commitments each country should strive to implement. Amongst these was a set of *Recommendations on Core Indicators for Rare Disease National Plans/Strategies* adopted unanimously by all MS on 6th of June 2013 (EUCERD 2013). The overall objective of these Recommendations was to enable the capturing of relevant data and information on the process of planning, implementing and monitoring of national plans/strategies. The resulting Core Indicators highlight important components for a robust and comprehensive national plan/strategy (Ferrelli et al. 2015).

## Establishing the status quo around national plans and strategies

The adoption of the EUCERD *Recommendations on Core Indicators for Rare Disease National Plans/Strategies* was accompanied by a commitment from Member States to regularly collect the information defined in a table within the body of the document. This was an important commitment, as the ability to share experiences and monitor activities of other countries (especially perhaps countries with similar geographies, health and social systems, languages, populations, etc.) appeared anecdotally to be of major benefit to the national plans/strategies goal. The Expert Groups for rare diseases were invaluable in this sense, as the traditional roundtables gave all countries a space (and indeed constituted an incentive) to provide updates on progress and reasons for any delay in adopting a plan/strategy. As the mandate of the Commission Expert Group on Rare Diseases expired in 2016, an alternative means of maintaining a pan-European overview was particularly important.

A logical vehicle for the collection of this data existed in the form of the Resource on the *State of the Art of Rare Disease Activities in Europe*, a well-established resource providing valuable, detailed information for all stakeholders in the field of rare diseases and orphan medicinal products. Under the EUCERD Joint Action (2012–2015), the report was produced annually by the Institut national de la santé et de la recherche médicale (INSERM), in 5 volumes, which were downloaded 15,000 times per year. Under RD-ACTION (2015–2018), production of the State of the Art moved to Newcastle University. The resource was streamlined and moved online, with several changes.

**Fig. 1:**
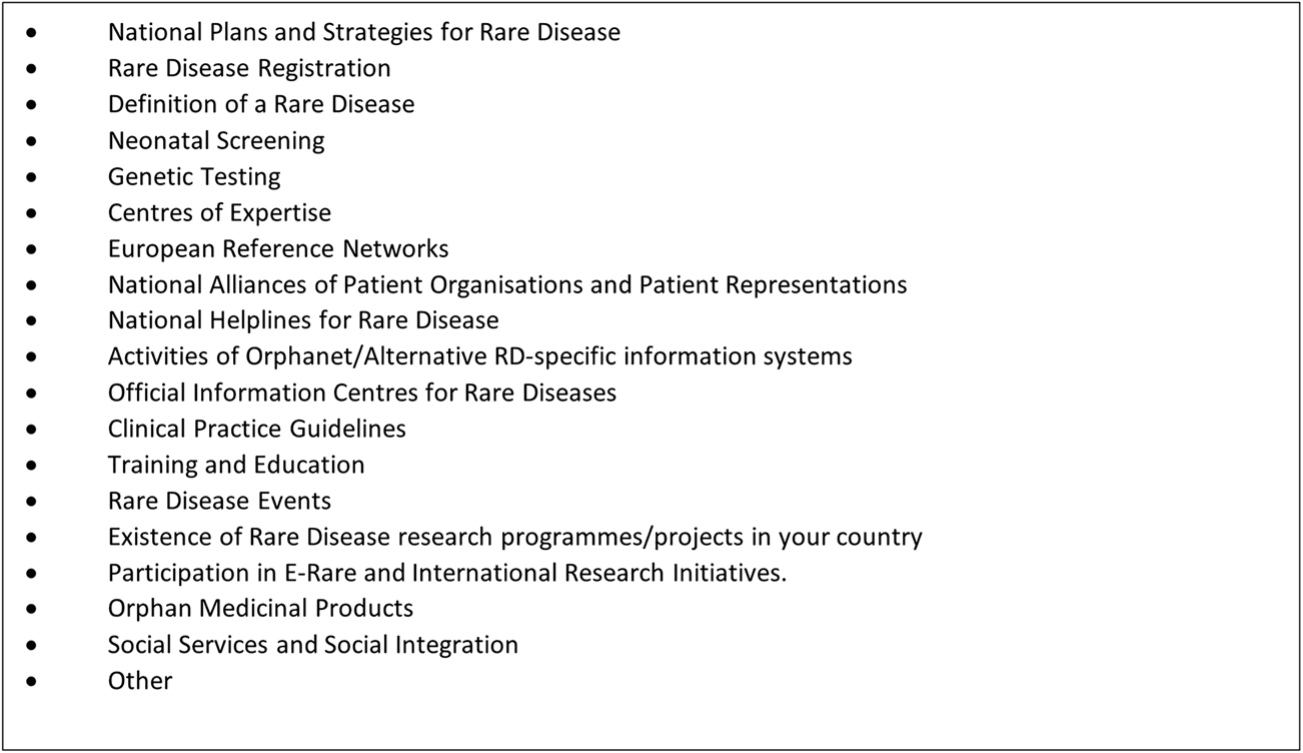
Structure of the survey used within the *Resource on the State of the Art of Rare Disease Activities in Europe*

Firstly, it was decided that the information elicited should be more harmonised and structured, to make the data more comparable; secondly, the data should be collected and presented in a more accessible and easily updateable format; and thirdly, a broader range of stakeholders should be consulted to provide the data on each country’s national activities, rather than relying on a single individual (initially, the MS representatives provided all information). To satisfy the latter, a ‘Data Contributing Committee’ (DCC) was created for each EU MS. These DCCs consist of representatives of the Competent National Authority (traditionally the official national representatives participating in the EUCERD and subsequent CEGRD), the National Alliance of RD patient organisations, and the national Orphanet team. In 2019, a new category of stakeholder was introduced to the DCC data requests namely the individuals representing each country in the Board of MS of ERNs. The intention is to try to ensure that by working together, the information submitted by each DCC constitutes an accurate and multistakeholder perspective on each topic, whilst reducing the workload for the National Competent Authority representatives. To increase the utility and comparability of the information collected from each country, a comprehensive online survey for the State of the Art resource was created, posing specific questions on many important aspects of a country’s RD activities (19 aspects at present—see Fig. 1).

**Fig. 2:**
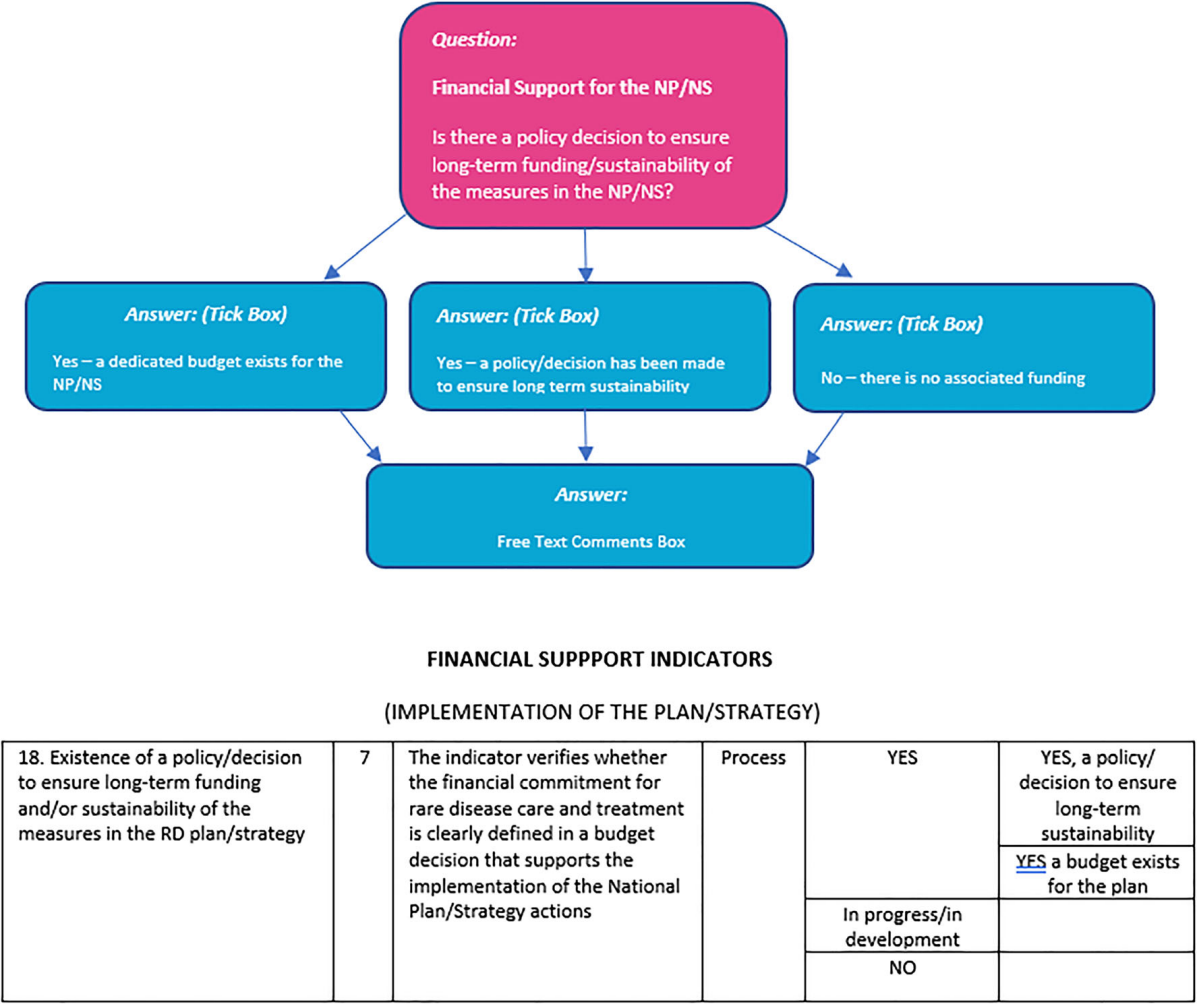
The structuring of the *State of the Art Resource* survey: the top image shows a question tree from the survey; the bottom image is part of the table from (EUCERD 2013)

Crucially, the survey questions were carefully constructed to yield comparable information to explore practices between countries. Many of these were directly based upon the aforementioned *EUCERD Recommendations on Core Indicators for Rare Disease National Plans/Strategies*, and provided the options proposed by these Recommendations (see for instance Fig. 2)

Since 2016, data has been requested at intervals from all EU MS, plus the UK (the intention is to expand the geographical reach, from 2021). The data is provided through a Lime Survey interface, on a token basis, meaning the members of each country’s DCC receive the same token and can work simultaneously and collaboratively to complete the various sections of the survey. Since 1st of January 2019, the core data collection and analysis aspects of the *Resource on the State of the Art of Rare Disease Activities in Europe* have been supported by the Rare 2030 Foresight Study (which is co-funded by the European Union Pilot Projects and Preparatory Actions Programme 2014-2020: PP-1-2-2018-Rare 2030).

Data provided in 2019 was utilised to elaborate and enrich a series of 8 Knowledge Base Summaries (https://www.rare2030.eu/knowledgebase/) constructed as part of the project’s broad consultation activities and intended to illustrate the status quo and summarise relevant initiatives and outputs (as a starting point to identify gaps and future needs). The data relating to national frameworks was extracted from the surveys and analysed to support this activity. It revealed that at Member State level, there is significant heterogeneity in the state of advancement of national policies, plans, or strategies for rare diseases (Hedley et al. 2019). Countries opted to address the challenge of the 2009 Council Recommendation in different ways. For instance, some countries have adopted a national strategy only, as opposed to adopting/following up with a plan (the usual conclusion being that a plan is composed of more specific, measurable actions, whereas a strategy may be more broad and high level). Most countries adopted policies which were in some way time-bound as opposed to open-ended. Some countries adopted national plans relatively early, but neglected to replace or refresh these when they expired. Others have not, to-date, met the stipulations of the Council Recommendation through adoption of a *specific* plan or strategy for rare diseases.

### The view from late 2020: results of a recent ‘State of the Art Resource’ data collection

**Fig. 3:**
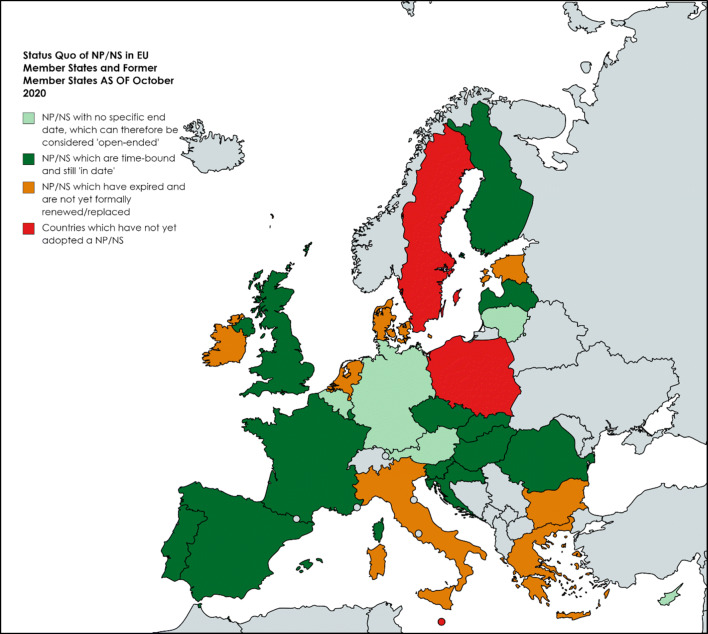
Status quo as regards national plans/strategies (NP/NS) for rare diseases, Oct 2020. Populated with data from the *State of the Art* resource and generated using mapchart.net

This map (Fig. 3) shows the status quo relating to national plans **as of October 2020**, following the most recent request to the Data Contributing Committees in all EU MS and former MS to review and update their data relating to NP/NS. Note that some countries have yet to provide full data to this resource, namely Poland and Greece. Other countries may update their data further in coming months, thus the picture presented here is based upon **the best data available through the** ***State of the Art*** **Resource**. NB UK data has been included in this analysis, as a former EU MS.

Twenty-five European MS/Former MS have adopted a National Plan or Strategy (NP/NS) for rare diseases at some stage, but unsurprisingly perhaps have tended to approach the mission in different ways and at different times (although most strived to meet the deadline of the end of 2013): the result is significant heterogeneity across Europe. Not all EU MS followed the recommendation to adopt a NP/NS for rare diseases by the end of 2013: Poland, Malta, and Sweden have yet to do so formally (Poland, it appears, is nearing approval of a first NP, and the most recent Swedish response notes that at present, rare diseases are named amongst the 25 National Program Areas). It is notable that the vast majority of the 25 countries which adopted a NP/NS at some stage opted to do so via a standalone policy document. Only Estonia opted to explicitly position a rare disease ‘Development Plan’ within the broader National Health Plan (nonetheless meeting the ‘alternative scenario’ stipulated under the Council Recommendation, Theme 1 a), namely to “explore appropriate measures for rare diseases in other public health strategies”).

The fact that 25 MS adopted a NP/NS for rare diseases at some point does not, by any means, equate to these nations having ‘live’ policies as of 2020. This is because most countries opted to adopt time-bound plans or strategies (as is more traditional perhaps, facilitating the setting of targets internally). In total, 20 countries have adopted time-bound national plans/strategies, with only Austria, Belgium, Cyprus, Lithuania, and Germany opting for essentially open-ended NP/NS (NB Austria initially adopted a time-bound plan 2014-2018, but evolved towards an open-ended approach. Germany is currently on the third ‘term’ (2018–2022) of its original plan which was adopted in 2013; therefore, for these purposes, it is deemed at present to be an open-ended plan, as it is not correct to say it has expired, nor has it been replaced by a new plan).

**Fig. 4:**
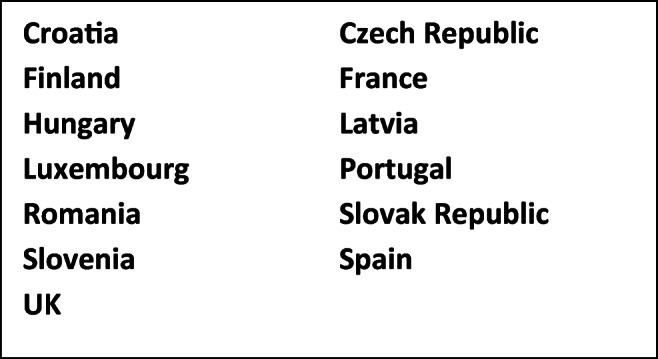
Countries with time-bound NP/NS ‘active’ as of October 2020

Of those 20, 13 have NP/NS which can be deemed ‘active’ of October 2020 (Fig. 4)

This means therefore that at the time of writing, 7 of the countries which opted to adopt a time-bound NP/NS *technically* no longer have active policies—the period defined for the plan/strategy has lapsed and there is no official renewal or adoption of a subsequent policy to continue the activities planned or required (which is not to say that activities are not continuing, of course). This is the case in Bulgaria (expired in 2013), Denmark (*apparently* expired 2019), Estonia (2017), Greece (2012), Ireland (2018), Italy (2016), and The Netherlands (2018). In some of the nations, efforts are underway to adopt a new NP/NS for rare diseases (e.g. Italy) but have not yet come to fruition (it is likely that COVID-19 disruption has caused significant delay, as in the Italian example).

Furthermore, it is clear that the existence of a national plan or strategy is one thing: implementation is quite another. There was always a danger that countries may elaborate and adopt reasonably robust and ambitious NP/NS for rare diseases, which, once approved—and the requests of the Council Recommendation of 2009 duly—fulfilled—would be largely overlooked and fade from public view. The *EUCERD Recommendations on Core Indicators for Rare Disease National Plans/Strategies* were largely concerned with establishing a NP/NS; however, some of the indicators can be used to gain a sense of the degree to which policies are actually being acted upon. An example of this is the existence (or otherwise) of a body specifically tasked with elaborating the NP/NS (in the pre-approval stage) and overseeing the implementation, once underway. Given the specificities of rare diseases, such bodies should be multistakeholder, involving at least clinicians, researchers, and patients, alongside professionals from across the health and social sphere. The latter is particularly important, as the Council Recommendation of 2009 specified that national plans and strategies should structure activities “within the framework of health and social systems”, and thus it would be logical to include representatives of not only ministries of health but also of social affairs, welfare, labour, employment, etc., to facilitate the requisite integration. It is acknowledged that rare disease populations are especially vulnerable and patients and families can face significant challenges in all aspects of life, in view of the rarity of the condition (EURORDIS 2017; EUCERD JA 2012): this necessitates specific measures to support people not only in obtaining more integrated and coordinated *medical* care but also to ensure a person-centred approach to care within a continuum, encompassing also the social, educational, and employment spheres (Castro et al. 2017; EURORDIS 2019).

The *State of the Art* Data Contributing Committees of the EU MS are asked, as part of their ‘national plans and strategies’ submission ‘Is there a dedicated body (expert advisory group) to oversee drafting or implementing of the NP/NS, or to evaluate the impact of the NP/NS?’ The phrasing leaves this open, to apply to the initial elaboration phase, the implementation phase, and, ideally once NP/NS are drawing to a close, an evaluation phase. The response here is multiple choice and reflects the goal of a well-functioning group, meeting regularly, with a multistakeholder composition (including patients).

The October 2020 data update within the scope of the *Resource on the State of the Art of Rare Disease Activities in Europe* yielded the following results*:*

Of the 18 NP/NS still ‘active’ (i.e. not expired) in EU MS/Former MS as of October 2020:

- A total of 10 reported that a ‘dedicated advisory body/expert advisory group’ of some sort is in place to oversee the implementation or evaluation of the Plan, and that this body was Multistakeholder **and** fully functioning (i.e. meeting regularly)
- A total of 6 reported that such an advisory body exists, is Multistakeholder, and is functioning (but not meeting regularly)
- A total of 2 reported a body which was ‘partially functioning but does not include all stakeholders’

Another important criterion by which to potentially assess the potency of NP/NS for rare diseases is the existence of financial support. As an additional survey question, countries are asked whether dedicated funding exists to support the implementation of the plan or strategy itself. Again, of the 18 NP/NS still ‘active’ (i.e. not expired) in EU MS/Former MS as of October 2020:

- A total of 14 reported **no dedicated funding behind the NP/NS (most state that actions contained within the NP/NS are funded through the general health system.** The 4 declaring that dedicated funding *was* associated with the plan itself were France (which specified funding for the Centres of Reference), Romania (just over 1.009 million Euros per year), Slovak Republic (240,000 Euros per year), and Belgium (which reported the sum of 15 million Euros per year).

## Conclusions and priorities in the post 2020 era

The collection of data via the *Resource on the State of the Art of Rare Diseases Activities in Europe* creates the possibility for cross-country analysis, illustrating the European status quo for a variety of rare disease-relevant topics, amongst them national plans and strategies. A potential downside, of course, is that the data is largely self-declared—furthermore, its accuracy relies upon DCC members dedicating significant time and energies to the completion of the survey. Reviewing this status quo from the vantage point of late 2020, the request from the Council to the EU MS in 2009 was, for the most part, fulfilled: whereas 5 MS had adopted a NP/NS for rare diseases in 2009, this figure has risen to 25. Moreover, the impact of this soft-legislation has not been limited to the political or geographical confines of the European Union alone. Of the non-MS EEA countries, Norway has adopted a national plan, as has non-EEA nation Switzerland. A number of EU candidate countries have either adopted a national plan or strategy (e.g. Serbia, Montenegro) or are in the process of doing so (The Republic of North Macedonia), and other nations in eastern Europe are advancing in this mission (e.g. Bosnia and Herzegovina have two connected plans, Ukraine is hoping to soon approve a NP/NS). And beyond Europe, more and more countries have, in the decade since the passage of the Council Recommendation of 2009, recognised the strategic advantages of adopting such a framework at national level (Dharssi et al. 2017; Hedley et al. 2018). The example and ambition of the European Union here has therefore been hugely influential.

However, this success must be tempered with caution: as of October 2020, **3 EU MS are lacking a first NP/NS and 7 have technically expired policies. Crucially, 2020 is the terminal point for many of the still-active plans and strategies**: Croatia, Czech Republic, Hungary, Latvia, Portugal, Romania, Slovak Republic, Slovenia, Spain, and UK will all see their current documents expire without rapid action over the next two months. Only France (now onto its 3rd national plan for rare diseases), Finland (which adopted its second plan spanning the years 2019–2024), and Luxembourg (2018–2022) will join the ‘open-ended’ plans/strategies of Austria, Belgium, Cyprus, Germany (see above), and Lithuania to have ‘live’ NP/NS as of January 2021. Furthermore, the impact of COVID-19 on nations worldwide is likely to push rare diseases further down the queue in terms of national priorities. This would be very damaging, potentially, as the COVID-19 has in fact already served to further illustrate the vulnerability of the rare disease population (many publications are expected in 2021, but see for instance Castro et al. 2021).

Since 2009, there has been no request or recommendation of similar weight from the European level to the MS to reflect upon, evaluate, and/or update or renew their NP/NS. Isolated recommendations have emerged in set of topic-specific recommendations adopted by the Expert Groups for Rare Diseases; for instance, the 2016 *Recommendations to Support the Incorporation of Rare Diseases into Social Services and Policies* (Commission Expert Group on Rare Diseases 2016) advises as follows:

*“1. The incorporation of RD specificities into mainstream social services and policies is a necessary element to be considered in future National Plans and Strategies (NP/NS) for RD and should be incorporated when existing NP/NS are evaluated and revised. In particular:*

- *Training of professionals should be promoted;*
- *High quality information should be made available****”***

Similarly, the 2015 *Recommendation on Cross-Border Genetic Testing for Rare Diseases* (Commission Expert Group on Rare Diseases 2015) stipulates that

*“1.1 The importance of adequate access to genetic testing for RD - including cross border genetic testing (CBGT) - when there is a clear clinical indication, should be stipulated in future National Plans and Strategies (NP/NS) for RD and should be incorporated when existing NP/NS are evaluated and revised”*

These examples notwithstanding, no rallying call to action has been made since the passage of the 2009 Council Recommendation on an action in the field of rare diseases, and thus momentum around NP/NS has, naturally, waned, for many stakeholders. Simultaneously, the expiration of the mandates for European Expert Groups for rare diseases has meant the removal of a suitable forum in which to unite competent national authority representatives tasked with addressing the myriad and complex challenges posed by rare diseases, and there is currently no multistakeholder body of comparable scale or standing to replace it. The Board of Member States of European Reference Networks (ERNs) offers limited opportunities for discussion of progress—or lack thereof—around NP/NS for rare diseases, but only insofar as this touches upon the ERNs which are the *reason d’* ê*tre* of this body: it does not have a mandate to address any and all issues relevant to rare diseases. A logical interface could be the urgent need to integrate ERNs—themselves a major success story of the past decade of European rare disease and specialised healthcare policy—to the national level, and indeed the Board of MS has highlighted the need to explore how ERNs and their national representatives could engage in national policy-making (Board of Member States of ERNs 2018). Such an analysis is very much-needed, as the national and European scenes have changed significantly since 2013, not least due to the creation of the ERNs themselves: renewed focus is needed on how to prepare and implement robust NP/NS for rare diseases which will be fit for the decade ahead, factoring in the need to strengthen national networks of healthcare—and social and holistic care—providers, to allow seamless integration of ERNs whilst enabling a bidirectional flow of knowledge, expertise, and data to ensure continued progress and meaningful outcomes for people living with rare diseases.

Besides the absence of an Expert Group or similar, there is no longer a project with the focus of EUROPLAN or the Joint Actions for Rare Diseases tasked with building capacity and developing resources at the European level, for adaptation and adoption at national level. An in-depth analysis of the extent to which NP/NS have in fact been implemented (as opposed to merely *existing* as relatively static documents), and the ways in which different countries have opted to orientate these documents, would be very valuable. The success or otherwise of particular approaches to, for instance, centre of expertise designation and networking, primary prevention (e.g. prevention of congenital anomalies), (Taruscio et al. 2014b), secondary prevention (e.g. newborn screening) and genetic testing, patient partnerships, rare disease registration and data capture, and support for the paramedical, social, and holistic needs of patients, would benefit nations seeking to make best use of increasingly-scant national resources whilst avoiding missteps of their forerunners. Even in terms of NP/NS methodological processes, there would be major advantages to greater cross-country collaboration: good practices on performing robust and independent evaluations of progress and identifying areas for improvement could be very useful to support the many countries whose original NP/NS have expired or will shortly do so.

From the vantage point of 2020, it is natural—and essential—to reflect critically upon the achievements of the past whilst looking to the next major horizon. The first of these has, to some extent, already taken place. In 2014, the Commission published an Implementation Report on both the Council Recommendation of 2009 and the Commission Communication of 2008 (European Commission 2014). It concluded that ‘by and large, the objectives of the Communication and the Council Recommendation have been reached’ but acknowledged that ‘there is still a long way to go’. The more recent report from the European Court of Auditors, however, highlighted the lack of concerted attention to the broad rare disease framework in Europe since this time, noting that ‘the Commission has not taken stock of its progress in the implementation of the EU rare disease strategy since 2014’ (European Court of Auditors 2019).

An important initiative in this quest to assess remaining gaps and areas of policy-need across Europe is the Rare 2030 Foresight Study. The project has built on the status quo across all aspects of rare disease diagnostics, treatment, care, research, and social support, to identify future-facing trends and rank these under the foresight methodology, to arrive at a number of contrasting future scenarios for the rare disease community in Europe in 2030 and beyond. Based upon considerations of preferability, plausibility, and possibility, the project is proposing a number of recommendations (Kole and Hedley 2021) to support the field in advancing towards the future its stakeholders most wish to see. The broad consultations undertaken across 2019 and 2020 highlight the need for renewed momentum, analysis, knowledge sharing, and guidance around the subject of national plans and strategies in particular. There is strong support for several activities here, including the following: evaluating the extent to which existing NP/NS have actually been implemented in European countries; encouraging and enabling countries to adopt their 2nd and 3rd NP/NS, particularly where 1st plans have lapsed; and defining key objectives and content for the NP/NS of the future—in cooperation this time with new actors, the ERNs—by identifying good practices which have yielded results in particular countries or regions, assessing their transferability to other countries/situations, and agreeing *new* issues and topics which should factor into the next generation of national policies. These brand new Rare 2030 recommendations (published in 2021) accord well with the messages of this paper and offer many tangible and practical calls for action.

In summary, the absence of both a suitable health and care-oriented forum—such as the former EUCERD or Commission Expert Group for Rare Diseases—*and* a project to support stakeholders in preparing, adopting, and implementing the next generation of national plans and strategies effectively means the momentum for countries to revisit these frameworks is arguably in a nadir, and the opportunities for robust cross-country problem-solving and sharing of experience are also in short supply. This is all the more concerning when one considers the unique importance of a robust national plan or strategy. In a world in which health and research-oriented issues of relevance to people with rare diseases risk becoming subsumed—and presumably diluted—under broader plans for health, genomics, cancer, and more, NP/NS should remain the primary vehicles to demonstrate the uniqueness of rare diseases and the impact they have on individuals, systems, and society at large (whilst ensuring appropriate *synergies* with aforementioned policies, to ensure no patient is left behind (Prades et al. 2020)). In the end, all needs pertaining to diagnostics, treatment, care, holistic wellbeing, and research should be addressed through robust national and supra-national solutions, detailed in an open and transparent way under the aegis of a strong national plan or strategy which all stakeholders in the field can stand behind.
